# Virtually the same? How impaired sensory information in virtual reality may disrupt vision for action

**DOI:** 10.1007/s00221-019-05642-8

**Published:** 2019-09-04

**Authors:** David J. Harris, Gavin Buckingham, Mark R. Wilson, Samuel J. Vine

**Affiliations:** School of Sport and Health Sciences, University of Exeter, St Luke’s Campus, Exeter, EX1 2LU UK

**Keywords:** VR, Haptic, Visually-guided, Dorsal, Ventral

## Abstract

Virtual reality (VR) is a promising tool for expanding the possibilities of psychological experimentation and implementing immersive training applications. Despite a recent surge in interest, there remains an inadequate understanding of how VR impacts basic cognitive processes. Due to the artificial presentation of egocentric distance cues in virtual environments, a number of cues to depth in the optic array are impaired or placed in conflict with each other. Moreover, realistic haptic information is all but absent from current VR systems. The resulting conflicts could impact not only the execution of motor skills in VR but also raise deeper concerns about basic visual processing, and the extent to which virtual objects elicit neural and behavioural responses representative of real objects. In this brief review, we outline how the novel perceptual environment of VR may affect vision for action, by shifting users away from a dorsal mode of control. Fewer binocular cues to depth, conflicting depth information and limited haptic feedback may all impair the specialised, efficient, online control of action characteristic of the dorsal stream. A shift from dorsal to ventral control of action may create a fundamental disparity between virtual and real-world skills that has important consequences for how we understand perception and action in the virtual world.

## Introduction

Despite the increasing popularity of virtual reality (VR) as a training tool in a range of industries, including sport, aviation and medicine, we know very little about the low-level perceptual effects of acting in a virtual world. Virtual reality is a collection of technologies that allow the user to interact with a simulation of some environment, in real-time, using their own senses and motor skills (Burdea and Coiffet 2003). Since the 1990s, VR has been adopted by psychological laboratories because it permits precise environmental control which can be untethered from the constraints of the physical world. This method has opened extensive experimental possibilities for the exploration of phenomena as diverse as the size-weight illusion (Buckingham 2019), allocentric memory (Serino et al. 2015) and movement-evoked pain (Harvie et al. 2015). In recent years, interest in the use of VR for a range of training purposes, including visually-guided motor skills, has also grown. Particular areas of application include surgery (Gurusamy et al. 2008), motor rehabilitation (Adamovich et al. 2009) and sport (Gray 2019). Visually guided skills such as these must be performed in a three-dimensional (3D) world, but the stereoscopic presentation of two-dimensional (2D) images in current head-mounted VR provides visual cues that have subtle, but important, differences from the real-world. It is not well understood how the unique perceptual environment of VR may influence how visually guided skills are performed and learned. In this short review, we highlight a number of findings which suggest visually guided action in the virtual world might differ substantively from the real-world. We propose that if fundamentally different modes of action control are activated in VR, skills performed in the virtual world will be unrepresentative of the real-world, and transfer of training will be compromised.

## Vision for action

Visual information for guiding real-time action is thought to be processed separately from more abstract perception in the mammalian brain, reflecting an evolutionary specialisation for the control of movement (Goodale 2017). The prevailing characterisation of visual processing identifies a ventral pathway (projecting from primary visual cortex, V1, to the inferior temporal lobe) that is primarily concerned with perception and identification of visual inputs, and a dorsal pathway (projecting from V1 to the posterior parietal lobe) which provides visual information for guiding real-time action (Goodale 2017; Goodale and Milner 1992; Milner and Goodale 1993). Vision-for-action and vision-for-perception pathways are separately susceptible to disruption from brain damage, indicating they are functionally segregated in the normal brain. Naturally, the two pathways interact on some level (Goodale and Cant 2007), but the dorsal pathway maintains a specialisation for visual control of skilled movement. There is a reason to question, however, whether this normal functional separation is maintained in the virtual world.

### Cues to depth in the virtual world

The primary reason vision for action may be disrupted in VR is the artificial presentation of depth information (Wann et al. 1995). Several findings have illustrated the impaired estimation of distance and a general perception of the virtual world as ‘flatter’, although this effect seems to attenuate in higher fidelity systems (Interrante et al. 2004, 2006). The dorsal stream relies primarily on binocular information (Mon-Williams et al. 2001), whereas monocular cues to distance (such as texture and perspective) tend to inform perceived distance through the ventral stream. Restricted binocular cues to depth do not preclude execution of visually guided tasks (Carey et al. 1998), but reliance on monocular cues does lead to increased use of the ventral stream for guiding action (Marotta et al. 1998) and, as a result, movement inefficiency (Loftus et al. 2004). The ventral stream is required for pre-planned or delayed movements but utilizes different information to guide action. If binocular cues are impaired in VR, as the general perception of ‘flatness’ suggests they might be, actions in the virtual world may be achieved using much greater ventral input than real-world skills.

The primary binocular cues to depth are binocular disparity and vergence. Vergence (the simultaneous horizontal rotation of the eyes to maintain binocular fixation) is an important cue to depth for the dorsal stream (Mon-Williams and Tresilian 1999; Mon-Williams et al. 2001). Perceived depth is constructed using a range of available cues, but Tresilian et al. (1999) propose that the weight afforded to vergence information decreases when there is a conflict between vergence and other depth cues—exactly as is the case in a VE. In the physical world, accommodation (the focusing of the lenses to maintain a clear image over distance) varies synchronously with vergence, but in head-mounted displays the normal connection is broken due to presentation of varying depth objects on a fixed depth screen (~ 5 cm from the eyes in head-mounted displays) (Eadie et al. 2000). This conflict may reduce the weight afforded to vergence as a cue to depth (Tresilian et al. 1999), leading to less reliable binocular information and a greater reliance on ventral processing (Marotta et al. 1998). Retinal image size also provides an effective cue to depth when object size is known. Lack of prior experience with and uncertainty about virtual objects may, however, make this cue uninformative as well. Consequently, general uncertainty about depth information may lead to a greater reliance on ventral mode control in VR.

Initial brain imaging findings have suggested that the normal pattern of dorsal and ventral activation may indeed be disrupted in VR. In the real-world, visual information about objects within arm’s reach (peripersonal space) tends to be encoded in the dorsal stream, while far-away objects (extrapersonal space) are processed using the ventral stream (Weiss et al. 2003). This reflects the archetypal dorsal/ventral distinction; near-by objects are potential targets for action, whereas far-away objects merely need to be recognised. To investigate this functional separation, Beck et al. (2010) asked participants to make spatial judgements about objects presented at near (60 cm) and far (150 cm) locations in virtual space. In contrast to the expected dissociation, fMRI indicated a disordered picture of dorsal and ventral activation, with near objects eliciting a high degree of ventral processing and far objects eliciting some dorsal activation. As discussed, visually guided motor skills can still be performed adequately with ventral mode control, (Loftus et al. 2004), but this finding raises concerns that visually guided actions in VR may operate through fundamentally different mechanisms to those performed in the real-world.

### Haptic information in the virtual world

An additional concern for the execution of visually guided motor skills in VR is the dearth of haptic information, which may also have negative effects on the user experience (Berger et al. 2018). Haptic feedback is derived from the active experience of touch but hand-held controllers in common VR systems do not change their tactile properties, other than providing vibrations to signal contact between virtual hands (or tools) and other surfaces. This kind of haptic information, however, remains unlike real-world feedback for most movements. Specialised feedback devices are currently being developed, such as haptic gloves and the Tesla full body suit, but extensive haptic feedback from exoskeleton-based systems remains expensive and impractical. There is reason to believe this general lack of haptic information may further push users into a ventral mode of processing, as has been observed for basic reaching and grasping movements (Goodale et al. 1994).

Terminal tactile feedback from target objects, which is absent in VR, is necessary for normal, real-time, reaching and grasping. Reaching to a virtual target (e.g. a mirror reflection or imagined target object) with no end-point tactile feedback has disruptive effects on grasp kinematics (e.g. the normally tight scaling between in-flight grip apertures with object sizes) indicative of a switch from real-time visual control (dorsal mode) to one dependent on cognitive supervision (ventral mode) (Goodale et al. 1994; Whitwell et al. 2015). A recent investigation by Wijeyaratnam et al. (2019) showed that when reaching to a target in a virtual environment (where the hand was represented by a cursor and no end-point feedback was present) movement kinematics were indicative of offline (i.e. ventral) control and impaired online corrective processes, even though visual feedback was available.

Such pantomimed reaching movements—those made to imagined, remembered or virtual targets which provide no endpoint feedback—are informative for understanding how the lack of haptic information may impact actions in VR. Pantomimed reaches to a target are made more slowly, reach a lower peak velocity and have lower movement amplitude due to inefficient ventral mode control (Goodale et al. 1994; Whitwell et al. 2015). Movements in VR are effectively pantomimed, as they provide no endpoint feedback, and accordingly are also slower and more exaggerated (Whitwell and Buckingham 2013). Taken together, the artificial presentation of visual depth cues, the peculiarities of haptic feedback, and the general uncertainty created by impoverished sensory information, seems likely to elicit a more ventral mode of control in VR than the real-world. If visually guided skills in VR do indeed rely on ventral mode control, even in part, skills learned or performed using these altered perceptual inputs may not be representative of their real-world counterparts.

## Other concerns

### Accommodating to accommodation

The accommodation-vergence conflict in VR also raises questions about how visual performance could be impaired following VR use, and how cues to depth might be un-learned. Initial findings have shown that immediately following VR use there may be a greater tolerance for accommodative and vergence error, leading to faster accommodation and vergence (Hackney et al. 2018), but impaired ability to maintain focus on a target (Mosher et al. 2018). Transient reductions in visual acuity have also been observed following just 10 min in a head-mounted VR system (Mon-Williams et al. 1993). As well as these immediate perceptual effects of VR, it is feasible that when learning skills which rely heavily on accommodation/vergence changes—such as target and aiming tasks which require shifting of gaze between the target and the projectile—the redundancy of cues such as accommodation could lead to a degree of unlearning. Analogous maladaptive aftereffects have been observed following conflict between optical flow and bodily inertia in VEs (see Wright 2014). If visually guided actions are learnt in VR where cues to depth differ from the outside world, alternative weightings of depth information could be acquired (e.g. Tresilian et al. 1999), leading to impaired transfer of training.

### Virtual bodies

A related issue that may be disruptive to the normal control of action is disembodiment in VR. Not only does the addition of a virtual body induce a greater sense of presence, but it influences distance estimation, a foundational input for action planning (Mohler et al. 2010). Gonzalez-Franco et al. (2019) found that in a blind walking task, where people typically underestimate distances by approximately 10% in virtual environments, the addition of a virtual body reduced the error, but only when users felt embodied. Further to this, a virtual body actually influences action control, improving stepping accuracy and lower limb coordination during obstacle avoidance (Kim et al. 2018). As such, inadequate representation of the physical body may be another barrier to realistic action control in virtual scenes.

### How real are virtual objects?

Finally, there may also be more fundamental concerns about how we interpret virtual objects as targets for action. For example, Snow and colleagues have illustrated important differences in brain and behavioural responses when viewing real objects, which afford the ability to act, and pictures of those same objects, which do not (Gomez and Snow 2017; Holler et al. 2019). Object images do not appear to activate action responses in dorsal stream motor networks in the same way as graspable real objects (Squires et al. 2016). What is currently unknown, however, is the extent to which objects in the virtual world provide affordances for action. For real-world objects, 3D volumetric characteristics and stereo cues inform the viewer of how it can be grasped, but the unusual way in which objects are interacted with in VR (i.e. using handheld controllers) may mean that this normal mode of interaction is disrupted. This was recently demonstrated by Linkenauger et al. (2015) who found that an embodied cognition effect, where reaching capability influences perceived distance, only took effect after participants became familiar with their reaching ability in VR. Indeed, changes in virtual arm size had no effect on perceived distance until participants had gained some experience reaching their target. Consequently, it is unknown whether or not a virtual tool might elicit responses that are more akin to a picture of a tool than a real one, especially when participants do not have direct prior experience with the virtual objects.

## Conclusions

In this brief review, we have raised a number of questions about how the novel perceptual environment and multisensory conflict experienced in VEs might substantively impact visually guided action. Unfortunately, it seems likely that many of these issues will remain despite the rapid advancement of VR technology. One problem that may be addressed in the near future is the vergence-accommodation conflict. Multifocal HMDs where multiple image planes are provided to span the viewer’s accommodation range are a potential solution, but currently require significant computing power (Mercier et al. 2017). Alternatively, advancements in augmented reality may soon be able to provide monocular focus cues that induce accommodation in line with eye vergence (Jang et al. 2017).

Nonetheless, the lack of realistic haptic information seems sure to be an ongoing issue. Devices such as haptic gloves and exoskeleton suits are able to provide rudimentary feedback, but they are unlikely to be sufficient for developing fine motor skills. More fundamental, is whether virtual entities are treated as real objects to act upon or more like pictorial stimuli. Advancing technologies are unlikely to address this issue. Additionally, some degree of sensory impairment, or at least uncertainty, seems likely to remain, all of which may contribute to fundamentally different modes of action control. It should be noted, however, that these issues only pertain to finely tuned, perceptual-motor abilities. As described by Slater (2009), virtual environments are able to elicit a range of realistic behavioural responses, such as actively avoiding illusory pits (Meehan et al. 2002) and maintaining social norms with virtual avatars (Sanz et al. 2015). The perceptual issues identified here are do not pose a problem for a range of behavioural outcomes such as these.

In light of the questions we have raised about the effect of impaired binocular cues on dorsal and ventral modes of processing, it may be informative for future work to investigate whether well-established signatures of dorsal/ventral control, measured through reaching and grasping kinematics, hold in VR (Ganel and Goodale 2003). Manipulating cues to depth in VEs may also prove instructive for understanding vision for action in virtual worlds, as well as addressing predictions of the perception–action model. As grasping kinematics for virtual or imagined targets appear to be qualitatively different (Goodale et al. 1994), it seems likely that other more complex actions might also diverge from the real skill. Overall, if different modes of visual processing are being engaged or different cues to depth are being relied upon, actions in VR may be more detached from real-world ones than we realise. Even if visually guided skills are performed adequately in VR, if a more ventral mode is being relied upon the skill is qualitatively different, which may have implications for transfer to real-world skills. These are important questions to address for the field of VR training and may help to explain when and why VR is an effective learning tool, and when it may be ineffective or even counterproductive.
